# Supplementary material for: Salivary Cortisol Reaction Norms in Zoo-Housed Great Apes: Diurnal Slopes and Intercepts as Indicators of Stress Response Quality
Source: Animals (Basel). 2022 Feb 21;12(4):522. doi: 10.3390/ani12040522 (PMC8868550; doi:10.3390/ani12040522)
Supplement: Supplementary file 1 [file animals-12-00522-s001.zip › animals-1546711-supplementary.pdf]

Supplementary Table S1: Number of salivary cortisol slopes and intercepts per individual.

			Old ape house	Enrichment days	New ape house	
Species	Age category	Sex	Number of slopes and intercepts			Total number
Orangutan	Adult	Male	1	10	1	12
		Female1	1	10	1	12
		Female2	1	10	1	12
		Female3	1	10	1	12
	Immature	Male1	1	10	1	12
		Male2	1	10	1	12
		Female	1	10	1	12
Bonobo	Adult	Male	1	10	1	12
		Female1	1	10	1	12
		Female2	1	10	1	12
		Female3	1	10	1	12
		Female4	1	8	1	10
		Female5	1	10	1	12
		Female6	1	10	1	12
	Immature	Male1	1	10	1	12
		Male2	1	10	1	12
		Male3	1	-	1	2
		Female1	1	10	1	12
		Female2	1	-	-	1
Gorilla	Adult	Male	1	10	1	12
		Female1	1	10	1	12
		Female2	1	7	1	9
		Female3	1	7	-	8
		Female4	1	6	1	8
		Female5	1	9	1	11
	Immature	Male1	1	10	1	12
		Male2	1	-	1	2
		Female	1	10	-	11
						**290**
